# Association between varicose veins and constitution of traditional Chinese medicine plus heart-failure-like symptoms

**DOI:** 10.3389/fcvm.2024.1465843

**Published:** 2024-10-23

**Authors:** Cheng-Ken Tsai, Oswald Ndi Nfor, Wen-Yu Lu, Yung-Po Liaw

**Affiliations:** ^1^ Department of Public Health and Institute of Public Health, Chung Shan Medical University, Taichung, Taiwan; ^2^ Division of Cardiovascular Surgery, Department of Surgery, E-Da Cancer Hospital, I-Shou University, Kaohsiung, Taiwan; ^3^ Department of Medical Imaging, Chung Shan Medical University Hospital, Taichung, Taiwan; ^4^ Institute of Medicine, Chung Shan Medical University, Taichung, Taiwan

**Keywords:** constitution of traditional Chinese medicine, chronic venous insufficiency, heart failure, heart-failure-like symptoms, varicose vein

## Abstract

**Background:**

Varicose veins are a common issue for employees in jobs that require prolonged standing compared with all other employees. However, its relationship with presentations of traditional Chinese medicine constitution is unknown. This study aimed to investigate their association.

**Material and methods:**

Data in the study were obtained from questionnaires of patients in Taiwan Biobank, enrolled from 2008 to 2020. The responses to the statement “I can see distorted blood vessels on my four limbs (varicose veins).” were categorized into none, mild, moderate, severe. and more severe, and the same scale was also used to classify breathing difficulties and hypotension.

**Results:**

A total of 11,293 participants were enrolled in the study. The prevalence of women was higher in the studied group compared with the control. Patients complained of breathing difficulties with moderate (30.49%) and severe discomfort (12.44%) in the diseased group. Regarding hypotension, 28.81% and 9.82% of the patients presented with moderate and severe hypotension, respectively. The cofactor odds ratio was 1.775 for severe breathing difficulty/moderate hypotension and 2.235 for severe breathing difficulty/severe hypotension, with statistical significance. The combined impact of breathing difficulties and hypotension increased with severity.

**Conclusions:**

Varicose veins had a higher association with breathing difficulties and hypotension as the severity of the condition worsened. The combined impact of breathing difficulties and hypotension increased as the disease progressed. Therefore, self-reported assessments can be a useful tool for evaluating patients with asymptomatic varicose veins before the development of “heart-failure-like symptoms” to reduce the risk of underdiagnosis.

## 1 Introduction

Varicose veins (VVs) are a common and important issue, with an incidence of 29.5%–39.0% in women and 10.4%–23.0% in men (1). The CEAP (Clinical-Etiology-Anatomy-Pathophysiology) clinical score is the most widely used tool to determine the severity of chronic venous insufficiency (CVI), with the range from C0 (absolutely no venous disease) to C6 (an open and active venous leg ulcer), and VVs are classified as C2 severity (2). Recent research indicated that females and people residing in urban areas were more susceptible to CVI, with the predominant age group being middle-aged adults, between 41 and 65 years (3). Moreover, various aspects related were associated with varicose veins, including marital status, weight and body mass index, also educational impacts. People who were married, had a normal body mass index, and possessed a moderate or higher educational background, were more likely to develop CVI (3). In addition, patient perception and beliefs about varicose veins, particularly among those who have inherited the condition, contributed to the progress of CVIs due to multifaceted factors (3). VVs, classified as C2 by the CEAP scale, are the most common issue of venous disease, followed by CVI, affecting more than 25 million people in the United States (4, 5). As a chronic venous disease, VVs occur in superficial veins with a diameter greater than 3 mm, and the vast major pathetic vessels are caused by damage to valves, leading to blood stagnation in the low-pressure vascular network vessels and resulting in varicose veins (6).

The constitution of traditional Chinese medicine is one of the oldest medical theories in traditional Chinese medicine (7). There is harmony and equilibrium within the human body (8, 9), and some fundamental substances, such as blood, body fluids, and spirit and vital organs, including the liver, spleen, lung, heart, and kidney, are closely associated and interacted with each other to form the body's constitution (7). Impaired blood circulation and dysregulation of fundamental substances lead to several physiological and psychological disorders (7). Various types of the constitution have been reported, such as physical muscular weakness, dizziness, weak voice, easily feeling tired, short breath, and sweating spontaneously (10, 11). The unspecified constitution symptoms have been noted to resemble the presentations of heart failure (12). However, the association between VVs and the constitution of traditional Chinese medicine symptoms, which could be “heart-failure-like,” remain unclear. This study aimed to investigate the relationship between VVs and the constitution of traditional Chinese medicine symptoms.

## 2 Materials and methods

### 2.1 Data resource

The data for this study were obtained from Taiwan Biobank. Taiwan Biobank is a population-based database that recruited volunteers from Taiwan aged 30–70 without a cancer history. The enrollment was from 2008 to 2020. All volunteers were required to sign an informed consent before enrollment. The information from physical examination, bioinformatics, and a questionnaire were collected during the recruitment procedure. Participants were enrolled after excluding the incomplete and missing data.

### 2.2 Definition of variables

In the Taiwan Biobank, VVs were assessed using a questionnaire. The responses were classified into five categories, as none, mild, moderate, severe, and more severe. In response to the statement “I can see distorted blood vessels on my four limbs (varicose veins),” we categorized the answer into no varicose veins (none) and with varicose veins (mild, moderate, severe, and more severe). Similarly, breathing difficulties and hypotension were categorized as no (none), moderate (mild), and severe (moderate, severe, and more severe). Moreover, the statement for breathing difficulties was, “I feel hard to breathe, need to breathe deeply” and hypotension was “I will feel the darkness in front of my eyes when I stand up suddenly.” Covariates in the models included gender (Women/Men), age (Age < 50/50 ≤ Age ≤ 70), vegetarian diet (No/Yes), cigarette smoking (No/Yes), alcohol intake (No/Yes), exercise (No/Yes), body mass index (BMI) (Normal weight/Underweight/Overweight Obesity), education level (Elementary and below/Junior and senior school/University and above), job type (non-prolonged standing/Prolonged standing), hypertension (No/Yes), and diabetes (No/Yes).

### 2.3 Ethics statement

This study was approved by the Institutional Review Board of Chung Shan Medical University Hospital (CSMUH No: CS1-20009). Taiwan Biobank participants provided written informed consent during enrollment.

### 2.4 Data analysis

The data was analyzed using SAS version 9.4 (SAS Institute, Cary, NC, USA). Patients were divided into two groups, with one cohort of patients without varicose veins (control cohort) and the other one of patients with varicose veins (diseased cohort). The logistic regression models were used to determine the relation of breathing difficulty, hypotension, and varicose veins. The results were presented as odds ratio (OR) and 95% confidence interval (95% CI). For demographic characteristics analysis, Student's *t*-test was used to examine the difference between varicose veins status and categorical variables (gender, age, breathing difficulty, hypotension, vegetarian diet, cigarette smoking, alcohol intake, exercise, BMI, job type, hypertension, and diabetes). The results were presented as numbers (n) and percentages (%). The significance level used for the analysis was 0.05.

## 3 Results

A total of 11,293 participants were enrolled in the study. The basic characteristics stratified by varicose vein status are reported in Table 1. VVs had a higher prevalence in women compared with men (82.32 vs. 17.68%). Among patients without varicose veins (control cohort), they reported no symptoms (*n* = 5,746, 69.08%), moderate (*n* = 1,954, 3.49%), and severe (*n* = 618, 7.43%) breathing difficulties. In a subgroup of patients with varicose veins (diseased cohort), 1,698 (57.08%), 907 (30.49%), and 370 (12.44%) patients presented with no, moderate, and severe discomfort, respectively. The proportion of moderate and severe symptoms of breathing difficulty was higher in the diseased cohort than in the control cohort (*P* < 0.0001). Regarding hypotension, there were 5,843 (70.25%), 2,001 (24.06%), and 474 (5.70%) patients presenting with no, moderate, and severe symptoms in the control group, respectively, while the diseased cohort had 1,826 (61.38%), 857 (28.81%), and (9.82%) patients the reported no, moderate, and severe hypotension, respectively. Hypotension was more common in the diseased compared to the control cohort (*P* < 0.0001).

**Table 1:** Basic characteristics of the study participants.

Variables	No varicose veins	Varicose veins	*P*-value
	(*n* = 8,318)	(*n* = 2,975)	
Breathing difficulty, *n* (%)			<0.0001
No	5,746 (69.08)	1,698 (57.08)	
Moderate	1,954 (23.49)	907 (30.49)	
Severe	618 (7.43)	370 (12.44)	
Hypotension, *n* (%)			<0.0001
No	5,843 (70.25)	1,826 (61.38)	
Moderate	2,001 (24.06)	857 (28.81)	
Severe	474 (5.70)	292 (9.82)	
Gender, *n* (%)			<0.0001
Women	4,579 (55.05)	2,449 (82.32)	
Men	3,739 (44.95)	526 (17.68)	
Vegetarian diet, *n* (%)			0.0440
No	7,461 (89.70)	2,629 (88.37)	
Yes	857 (10.30)	346 (11.63)	
Age, *n* (%)			0.0176
Age <50 (ref)	4,134 (49.70)	1,554 (52.24)	
50≤ Age ≤70	4,184 (50.30)	1,421 (47.26)	
Cigarette smoking, *n* (%)			<0.0001
No	6,537 (78.59)	2,692 (90.49)	
Yes	1,781 (21.41)	283 (9.51)	
Alcohol intake, *n* (%)			<0.0001
No	7,550 (90.77)	2,837 (95.36)	
Yes	768 (9.23)	138 (4.64)	
Exercise, *n* (%)			0.0003
No	4,814 (57.87)	1,834 (61.65)	
Yes	3,504 (42.13)	1,141 (38.35)	
BMI categories, *n* (%)			<0.0001
Normal weight (18.5 ≤ BMI < 24 kg/m^2^)	4,002 (48.11)	1,688 (56.74)	
Underweight (BMI < 18.5 kg/m^2^)	231 (2.78)	80 (2.69)	
Overweight (24 ≤ BMI < 27 kg/m^2^)	2,381 (28.62)	748 (25.14)	
Obesity (BMI ≥ 27 kg/m^2^)	1,704 (20.49)	459 (15.43)	
Education level *n*, %			0.0809
Elementary and below	387 (4.65)	125 (4.20)	
Junior and senior school	3,291 (39.56)	1,244 (41.82)	
University and above	4,640 (55.78)	1,606 (53.98)	
Job type *n*, %			<0.0001
Non-prolonged standing	3,307 (39.76)	1,058 (35.56)	
Prolonged standing	5,011 (60.24)	1,917 (64.44)	
Hypertension, *n* (%)			<0.0001
No	6,679 (80.30)	2,568 (86.32)	
Yes	1,639 (19.70)	407 (13.68)	
Diabetes			<0.0001
No	7,542 (90.67)	2,793 (93.88)	
Yes	776 (9.33)	182 (6.12)	

The association between breathing difficulties and VVs was determined using logistic regression (Table 2). When using no breathing difficulty as the reference group, the OR of moderate breathing difficulty was 1.357 (95% CI = 1.228–1.499) and 1.623 (95% CI = 1.403–1.877) for severe breathing difficulty, with statistical significance in *P* trend test (*P* < 0.0001). Moreover, the result of the association between hypotension and VVs is shown in Table 3. The OR of moderate hypotension was 1.283 (95% CI = 1.159–1.420) and 1.659 (95% CI = 1.409–1.953) in severe hypotension when compared with patients without hypotension. The trend was statistically significant (*P* < 0.0001).

**Table 2:** The association between breathing difficulty and varicose veins.

Variables	OR	95% CI	*P*-value
Breathing difficulty			
No (ref)	–		
Moderate	1.357	1.228–1.499	<0.0001
Severe	1.623	1.403–1.877	<0.0001
*P* for trend	*P*-value <0.0001		
Gender			
Women (ref)	–		
Men	0.307	0.272–0.347	<0.0001
Vegetarian diet			
No (ref)	–		
Yes	1.003	0.874–1.151	0.9709
Age			
Age < 50 (ref)	–		
50 ≤ Age ≤ 70	1.061	0.961–1.171	0.2396
Cigarette smoking			
No (ref)	–		
Yes	0.791	0.677–0.926	0.0034
Alcohol intake			
No (ref)	–		
Yes	0.974	0.794–1.196	0.8041
Exercise			
No (ref)	–		
Yes	0.928	0.845–1.019	0.1182
BMI categories			
Normal weight (ref)	–		
Underweight	0.697	0.534–0.910	0.0080
Overweight	0.992	0.892–1.103	0.8822
Obesity	0.883	0.777–1.004	0.0575
Educational level			
Under elementary school (ref)	–		
Junior and senior school	1.280	1.026–1.597	0.0290
University and above	1.340	1.069–1.679	0.0110
Job type			
Non-prolonged standing (ref)	–		
Prolonged standing	1.279	1.167–1.402	<0.0001
Hypertension			
No (ref)	–		
Yes	0.859	0.754–0.978	0.0219
Diabetes			
No (ref)	–		
Yes	0.802	0.669–0.960	0.0160

**Table 3:** The association between hypotension and varicose veins.

Variables	OR	95% CI	*P*-value
Hypotension			
No (ref)	–		
Moderate	1.283	1.159–1.420	<0.0001
Severe	1.659	1.409–1.953	<0.0001
*P* for trend	*P*-value <0.0001		
Gender			
Women (ref)	–		
Men	0.294	0.261–0.333	<0.0001
Vegetarian diet			
No (ref)	–		
Yes	1.028	0.896–1.180	0.6898
Age			
Age <50 (ref)	–		
50≤ Age ≤70	1.086	0.983–1.199	0.1048
Cigarette smoking			
No (ref)	–		
Yes	0.801	0.685–0.937	0.0054
Alcohol intake			
No (ref)	–		
Yes	0.983	0.801–1.206	0.8680
Exercise			
No (ref)	–		
Yes	0.916	0.834–1.006	0.0654
BMI categories			
Normal weight (ref)	–		
Underweight	0.689	0.528–0.899	0.0061
Overweight	1.011	0.909–1.125	0.8335
Obesity	0.918	0.808–1.043	0.1889
Educational level			
Under elementary school (ref)	–		
Junior and senior school	1.281	1.027–1.598	0.0280
University and above	1.336	1.066–1.673	0.0118
Job type			
Non-prolonged standing (ref)	–		
Prolonged standing	1.279	1.167–1.402	<0.0001
Hypertension			
No (ref)	–		
Yes	0.879	0.772–1.001	0.0525
Diabetes			
No (ref)	–		
Yes	0.804	0.672–0.962	0.0172

Furthermore, the results of logistic regression analysis of the association between breathing difficulties, hypotension, and VVs are illustrated in Table 4. When taking patients with no breathing difficulty/no hypotension as a reference, the cofactor OR of patients with no breathing difficulty/moderate hypotension was 1.186 (95% CI = 1.033–1.361), while no breathing difficulty/severe hypotension was 1.357 (95% CI = 1.030–1.788). Furthermore, the cofactor OR of moderate breathing difficulty/no hypotension was 1.264 (95% CI = 1.110–1.440), moderate breathing difficulty/moderate hypotension was 1.582 (95% CI = 1.359–1.842), and 1.970 (95% CI = 1.514–2.563) in moderate breathing difficulty/severe hypotension. Additionally, the cofactor OR was 1.440 (95% CI = 1.155–1.796) for patients with severe breathing difficulty/no hypotension, 1.775 (95% CI = 1.403–2.246) for severe breathing difficulty/moderate hypotension, and 2.235 for severe breathing difficulty/severe hypotension (95% CI = 1.689–2.957).

**Table 4:** The association between breathing difficulty, hypotension and varicose veins.

Variables	OR	95% CI	*P*-value
No breathing difficulty, no hypotension	–		
No breathing difficulty, moderate hypotension	1.186	1.033–1.361	0.0157
No breathing difficulty, severe hypotension	1.357	1.030–1.788	0.0302
Moderate breathing difficulty, no hypotension	1.264	1.110–1.440	0.0004
Moderate breathing difficulty, moderate hypotension	1.582	1.359–1.842	<0.0001
Moderate breathing difficulty, severe hypotension	1.970	1.514–2.563	<0.0001
Severe breathing difficulty, no hypotension	1.440	1.155–1.796	0.0012
Severe breathing difficulty, moderate hypotension	1.775	1.403–2.246	<0.0001
Severe breathing difficulty, severe hypotension	2.235	1.689–2.957	<0.0001
Gender			
Women (ref)	–		
Men	0.309	0.274–0.350	<0.0001
Vegetarian diet			
No (ref)	–		
Yes	1.013	0.883–1.163	0.8536
Age			
Age <50 (ref)	–		
50≤ Age ≤70	1.103	0.998–1.218	0.0551
Cigarette smoking			
No (ref)	–		
Yes	0.787	0.673–0.921	0.0028
Alcohol intake			
No (ref)	–		
Yes	0.974	0.794–1.196	0.8020
Exercise			
No (ref)	–		
Yes	0.931	0.848–1.023	0.1372
BMI categories			
Normal weight (ref)	–		
Underweight	0.688	0.527–0.899	0.0061
Overweight	1.007	0.905–1.120	0.9049
Obesity	0.905	0.796–1.029	0.1290
Educational level			
Under elementary school (ref)	–		
Junior and senior school	1.265	1.014–1.579	0.0374
University and above	1.313	1.048–1.646	0.0180
Job type			
Non-prolonged standing (ref)	–		
Prolonged standing	1.280	1.167–1.403	<0.0001
Hypertension			
No (ref)	–		
Yes	0.873	0.767–0.995	0.0416
Diabetes			
No (ref)	–		
Yes	0.801	0.669–0.959	0.0159

## 4 Discussion

There are shared risk factors between VVs and cardiovascular diseases, including obesity, aging, smoking, and diabetes. Both conditions also share the pathophysiology of endothelial dysfunction, inflammation, and thrombosis (13). Diagnosis of both heart failure and chronic venous disease rely on typical physical examination; however, given that leg edema was the most common manifestation of CVI, there could be confusion or coexistence between VVs and undetected heart failure. Risk factor prediction is extremely important to enhance the diagnosis in VV patients with “hear-failure-like symptoms” or the so-called “Constitution of Traditional Chinese Medicine.” The present study demonstrated that females had a higher prevalence of VVs, and prolonged standing jobs were more prevalent in patients with VVs compared with the control group (Table 1). The proportion of moderate and severe symptoms of breathing difficulties and hypotension were more common in the VV cohort. The combined impact of breathing difficulties and hypotension increased with severity (Table 4).

A study including 10,423 adult participants used self-reported venous symptoms to define the CEAP and classify CVI (14). Compared with men, women had a higher burden of chronic venous disease, which is compatible with the results in the present study, with a higher prevalence of VVs in women compared with men (82.32 vs. 17.68%) (14). The present study also reported that being male was a negative risk factor compared with being female (OR = 0.309, 95% CI = 0.274–0.350, *P* < 0.001, Table 4). Prior studies have explored the risk factors for CVI, including age, female sex, and smoking (15–17), which supported the findings in the present study (Table 1). Obesity affects up to one-quarter of lower limb venous disease, is an important risk factor for VVs, and is more likely to be symptomatic because of their lower limb venous disease (18, 19). However, there was no remarkably higher prevalence of obesity in the diseased group compared to the control (Table 1). The reporting bias could be due to the invisibility of distorted blood vessels on the limbs of patients with obesity when the study was based on self-reported assessments. The present study reported that jobs needing prolonged standing were more common in patients with VVs (Table 1), which is consistent with a previous study showing a higher risk of VV of employees with jobs requiring prolonged standing compared with other employees (1.75 for men and 1.82 for women) (20). Diabetes is a risk factor for VVs (21–23); however, antidiabetic medication, such as metformin, could reduce the risk of VVs in patients with diabetes (24). The present study also demonstrated that diabetes was a negative predictor for VVs (OR = 0.801, 95% CI = 0.669–0.959, *P* = 0.0159, Table 4).

There were two main symptoms of the constitution of traditional Chinese medicine in this study, including breathing difficulty and hypotension. The association between each symptom with VVs varied according to its severity. (1) If patients presented with only one symptom, the contribution of breathing difficulty was higher than hypotension in the association with VVs (if there was no breathing difficulty, the cofactor OR of moderate hypotension = 1.186; severe hypotension = 1.357, while cofactor OR of moderate breathing difficulty = 1.264 and severe breathing difficulty = 1.440 when there was no hypotension). (2) If one of the two symptoms was moderate in severity, the cofactor of hypotension had a greater association with VVs than with breathing difficulties (under moderate breathing difficulty situations, respective cofactor ORs of moderate and severe hypotension = 1.582 and 1.970, respectively, while under moderate hypotension situation, cofactor ORs of moderate and severe breathing difficulty = 1.583 and 1.775, respectively, Table 4). However, (3) If one of the two symptoms was severe, the cofactor of breathing difficulties was more associated with VV than hypotension (under severe breathing difficulty situation, respective cofactor ORs of moderate and severe hypotension were 1.775 and 2.235, respectively, while under severe hypotension situation, cofactor ORs of moderate and severe breathing difficulty = 1.970 and 2.235, respectively, Table 4). Moreover, when patients complained of both symptoms, the risk of VVs was higher if the severity of any one of the symptoms was worse.

Previous evidence demonstrated a higher prevalence of clinical conditions of cardiovascular comorbidities (such as atrial fibrillation, congestive heart failure, and myocardial infarction) as CVI progressed (25). The reason for the variant association between symptoms and VVs could be explained by the insufficient preload and the complications of stationary flow in VVs. As VVs progressed, reduced venous return to the heart and pooling of blood below the inferior vena cava led to hypotension, tachycardia, and lower extremity edema (26). As the severity gets worse, venous stasis or turbulent flow predisposes to thrombotic events (27). Previous evidence has indicated that a history of deep vein thrombosis (DVT) increases the incidence of VVs with a hazard ratio of 2.6 (14, 15). Moreover, a retrospective cohort study investigating >400,000 patients in Asia demonstrated that VV increased the incidence of DVT drastically with a hazard ratio of 5.3 (28). Additionally, patients with VVs had a higher incidence of DVT than the control group (6.55 vs. 1.23 per 1,000 person-years) (28). In a study with 1.2 million people, 40% of patients had pulmonary embolism with or without DVT (29), As one criteria of Virchow's triad, venous stasis contributes to thromboembolic events and pulmonary embolism. The present study reported that breathing difficulties revealed a tighter association with VVs compared with hypotension when there were severe symptoms, which could be attributed to coexisting impacts from inadequate preload and potential thromboembolic events.

There was a 36.5% prevalence of chronic venous diseases in patients with any cardiovascular disease, with the highest prevalence of 30% in patients with lower extremity edema and the lowest prevalence in patients with ulceration (13). However, even in C3–C6 severity, 2.8–32.2% of patients were symptomatic, while 14.0–37.6% of patients were asymptomatic. In C2 patients, there were only 1.2%–4.9% symptomatic patients, compared with 6.4%–8.8% asymptomatic patients (25). As the patients were younger, more asymptomatic patients were reported, which could lead to underdiagnosis of VVs (25). Less than 50% of patients were older than 50 years in the present study, and the prevalence of asymptomatic patients with VV may mask the accurate diagnosis of VVs. In addition to congestive heart failure and pulmonary hypertension, VVs have also been characterized by unspecified “heart-failure-like symptoms,” such as fatigue, dizziness, anorexia, palpitations, and shortness of breath on exertion (26). Self-report by self-observation could elevate the diagnosis of VVs in the early stages before symptoms develop. In terms of therapy, intermittent use or full compliance of compression stockings have been recommended by European Society for Vascular Surgery as one of the conservative treatment modalities for chronic venous disease (30). It has been reported to be effective in relieving symptoms in patients with chronic venous disease by decreasing pain, heaviness, cramps, and oedema (30). Unfortunately, the present study has opted for a questionnaire approaching database with self-reported venous assessment, which was lacking in the documentation on whether elastic compression stockings were used for symptom relief.

Since VVs disease has been a crucial issue, many evidences have reported that chronic venous disease is relevant to cardiovascular disorders (30). Patients are prone to developing venous diseases and symptoms, such as edema, leg pain, and cutaneous changes. Therefore, history-taking and physical assessment are clinchers to identify coexisting cardiovascular diseases (30). Moreover, patients with VVs have two-fold increase in the risk of major adverse cardiovascular events, including congestive heart failure, ischaemic stroke, DVT and pulmonary embolism (30). Fortunately, both Wells score and D-dimer levels could be considerable options used to evaluate the possibility of venous thrombosis and the necessity for advanced diagnostic imaging such as ultrasonography or computed tomography venography (31). Furthermore, recent article also indicated the gene correlation between varicose vein and heart failure, suggesting that people with varicose veins might have a higher risk of heart failure (31). One practical hint of the edema relevant to CVI is pitting pattern, originating in the peri-malleolar region, progresses up the leg in a dependent manner but spares the forefoot (32). Since more and more evidences have highlighted the crosstalk between VVs and heart failure, self-report assessment focusing on leg is a simple and quick check-up regarding the concerns of both VVs, early heart failure sign, or concomitant events.

## 5 Strengths and limitations

Despite limitations of lacking of objective information, such as electrocardiography, echocardiography, or biochemistry data, the present study gathered objective questionnaire data from a substantial cohort of 11,293 participants. This approach offered an efficient and practical tool that closely mimicked real-world applications for both primary care providers and specialists. This study reinforced the idea of educating patients and clinical practitioners to observe their legs, and further useful information could be acquired to reduce the risk of thrombosis and avoid misdiagnosis of other cardiovascular diseases.

## 6 Conclusions

VVs had a tight association with breathing difficulty and hypotension as the disease progressed, and the combined impact of the two symptoms also increased their association with VVs as the severity increased. For asymptomatic patients with VVs, it can be a simple method to assess the severity of VV by self-reported observation before it progresses to “heart-failure-like symptoms.”

## Data Availability

The datasets presented in this article are not publicly accessible due to restrictions on their usage. Requests for data access should be directed to Cheng-Ken Tsai at m871373@gmail.com.
